# Dental implant-associated oral squamous cell carcinoma: a clinical retrospective study of 22 cases

**DOI:** 10.1186/s40902-025-00490-9

**Published:** 2025-10-29

**Authors:** Jin Seok Kim, Young Heon Jeong, Heonwoo Lee, Kang-Min Ahn

**Affiliations:** 1 Department of Oral and Maxillofacial Surgery, College of Medicine, University of Ulsan, Asan medical center, Seoul, Korea, Republic of; 2 Department of Pathology, College of Medicine, University of Ulsan, Asan medical center, Seoul, Korea, Republic of

**Keywords:** Oral squamous cell carcinomas, Dental Implantation, Peri-implantitis

## Abstract

**Background:**

In oral cavity, oral squamous cell carcinoma (OSCC) is the most common malignant neoplasm. Established risk factors for OSCC include tobacco use, genetic predisposition, viral infections, and poor oral hygiene. With the increasing prevalence of dental implant (DI) procedures, reports of OSCC developing around implants have also risen. This study aims to investigate potential risk factors and clinical characteristics of OSCC occurring in association with dental implants, in order to improve understanding of its etiology and prognosis.

**Methods:**

This retrospective study analyzed the medical records of 22 patients diagnosed with OSCC around dental implants at the Department of Oral and Maxillofacial Surgery at a single institution, between 2009 and 2024 by a single surgeon. All patients presented with persistent discomfort or abnormal lesions following implant placement and were subsequently diagnosed with OSCC via histopathological examination. Pre-treatment imaging included magnetic resonance imaging, contrast-enhanced computed tomography, PET/CT, and bone scans. The 8th edition of the American Joint Committee on Cancer (AJCC) guidelines was used to determine cancer staging. Data were collected on patient demographics, smoking and alcohol histories, implant placement date, and histopathologic findings through electronic medical records.

**Result:**

All 22 patients presented with inflammatory lesions or progressive bone loss around dental implants. Five patients had a history of malignancy in other organs or hematologic disease. Six male patients had habitual alcohol consumption and smoking. OSCC occurred predominantly in the mandible (77.3%), with 95.5% of tumors classified as T4 at diagnosis. Neck metastasis developed in 41% of patients (9/22), including four cases of delayed/occult nodal involvement. The mean interval from implant placement to OSCC diagnosis was 4.7 years, with nearly half diagnosed within 1 year. Histopathology revealed only well- or moderately differentiated tumors, with bone invasion present in 95.5% of cases. The mean depth of invasion was 13.2 mm. Twenty patients underwent surgical resection, while two received nonsurgical treatment due to systemic condition. The mean survival time was 25.7 months, and Kaplan–Meier analysis showed no significant survival difference between maxillary and mandibular OSCC.

**Conclusion:**

OSCC after dental implant placement, though uncommon, is a significant clinical issue due to its late diagnosis, aggressive bone invasion, and poor prognosis. Our findings underscore the importance of differentiating OSCC from peri-implantitis and recommend prompt biopsy when peri-implant inflammation persists despite conventional treatment. Early recognition may improve survival outcomes. The observed links with prior malignancy, chronic inflammation, and lifestyle risk factors suggest a multifactorial etiology, highlighting the need for further longitudinal studies to refine prevention and early detection strategies.

## Background

Oral squamous cell carcinoma (OSCC), arising from the squamous epithelial cells in the oral cavity, represents more than 90% of all oral cancers [1, 2]. The most commonly affected sites include the floor of the mouth, tongue, gingiva, and buccal mucosa [3]. Clinically, OSCC often presents as a non-healing ulcer with indurated borders, a mass, or erythematous and leukoplakic lesions [4, 5]. In its early stages, OSCC may be asymptomatic; however, as the disease progresses, symptoms such as soft tissue swelling, pain, tooth mobility, and bleeding may emerge. Several risk factors are known to contribute to the development of OSCC, including alcohol and tobacco use, human papillomavirus (HPV) infection, prolonged exposure to chronic inflammation, inadequate nutrition, and genetic predisposition [6–8].

Dental implant (DI) surgery is now a commonly used and favored method for replacing missing teeth. However, with the increasing prevalence of DI placement, the incidence of dental implant related complications has also risen [9]. Among these, peri-implantitis, an inflammatory condition associated with progressive bone loss and soft tissue swelling, is the most frequently reported complication [10, 11]. Peri-implantitis typically presents with clinical signs such as erythema, edema, tissue hypertrophy, and, in some cases, ulceration of the surrounding soft tissue. These manifestations can closely mimic malignant lesions, emphasizing the importance of a careful differential diagnosis [12]. According to a systematic review and meta-analysis by Derks et al., the prevalence of peri-implantitis is estimated to range from 14 to 30% [10]. Initial treatment strategies generally focus on infection control using conservative approaches, including implant surface decontamination and antiseptic agents [13, 14]. In cases where these measures fail and marginal bone loss is evident, surgical debridement or implant removal may be necessary. Notably, approximately 35% of implant failures occur within the first year following surgery [11].

As the number of DI placements continues to increase, the incidence of such malignancies may also rise [15]. In recent years, several case reports have documented the occurrence of malignant tumors developing adjacent to dental implants. Oh et al. reported two such cases of malignant tumors arising around dental implants, with malignancies appearing within months to 1 year after implant placement [15]. Similarly, Kaplan et al. reviewed 25 studies comprising 47 cases of malignancies arising around dental implants, 40 of which were diagnosed as OSCC; the latency period in their review ranged from several months to 13 years, with most cases occurring within the first year [16]. These findings suggest that implant-associated OSCC may not be as rare as previously thought, but variability in latency highlights the need for further clarification.

In this study, we retrospectively analyzed the medical records of 22 patients who presented to our clinic with persistent discomfort or inflammatory lesions following dental implant placement and were subsequently diagnosed with OSCC. The purpose of this investigation is to characterize implant-associated OSCC by examining its risk factors, histopathological features, and clinical presentation, thereby contributing to a better understanding of this emerging clinical entity.

## Methods

### Patients

This retrospective study, approved by the Institutional Review Board (IRB No. S2025-0448–0001), analyzed the clinical and pathological data of 22 patients who presented to a single surgeon at the Department of Oral and Maxillofacial Surgery in a single institution between 2009 and December 31, 2024. All patients had previously undergone dental implant surgery at other institutions or private clinics. They were referred to the department when abnormal lesions were detected around a dental implant or after implant removal performed for persistent pain or progressive bone loss. All patients were subsequently diagnosed with OSCC based on histopathological examination. Inclusion criteria comprised primary OSCC arising from the gingiva or alveolar bone directly surrounding a dental implant, with the tumor either encircling or centered on the implant fixture. Tumors that primarily originated at natural teeth—with carcinoma centered on the tooth and secondarily extending to an adjacent implant—were excluded.

All patients underwent comprehensive imaging for cancer diagnosis, including magnetic resonance imaging (MRI), contrast-enhanced computed tomography (CT), positron emission tomography/computed tomography (PET/CT), and bone scans. These evaluations confirmed that the tumors arising around the dental implants represented primary OSCC rather than metastatic disease or local recurrence. Cancer staging was performed according to the 8th edition of the American Joint Committee on Cancer (AJCC) guidelines [17, 18].

Clinical information was obtained from electronic medical records and encompassed the following parameters: age, gender, smoking and alcohol consumption history, relevant medical history, implant placement date, tumor location, surgical treatment records, and histopathological findings. The dental implant models could not be identified due to limited information from other institutions.

### Diagnosis and surgical procedures

Patient data, including medical records, clinical photographs, and radiologic imaging (panoramic radiographs, enhanced CT, MRI, PET/CT, and bone scans) was comprehensively reviewed for diagnostic evaluation. Of the 22 patients, 20 underwent surgical treatment at a single institution. Two patients, owing to poor systemic condition, received palliative radiotherapy or neoadjuvant chemoradiotherapy instead. Based on tumor extent and the presence or absence of cervical lymph node metastasis, patients received either primary tumor resection alone or resection combined with neck dissection. In selected cases, reconstruction with a free vascularized flap was also performed.

### Histopathology

Excised surgical specimens during surgery were submitted to the Department of Pathology at the single institution for final histopathological analysis. Histopathological evaluation included cell differentiation, depth of invasion (DOI), perineural invasion (PNI), lymphovascular invasion (LVI), worst pattern of invasion (WPOI), neck metastasis, HPV test, and bone invasion. Formalin-fixed paraffin-embedded tumor tissue was harvested, and high-risk HPV types [16, 18, 33, 31, 53, 58, 35] and low-risk types [6, 11] were detected using a laboratory-developed PCR assay validated by the Molecular Diagnostic Laboratory, Department of Pathology at the single institution. HPV testing results were unavailable for 11 cases because the specimens had not been processed for HPV at the time of diagnosis. DOI could not be determined in four patients: two did not undergo surgery due to their poor general condition. In the other two cases, OSCC was not initially suspected, and sequestrectomy was performed under the impression of osteomyelitis (one at an outside facility and the other at the institution). Both specimens were later diagnosed as SCC, but DOI could not be determined from the limited tissue. Final cancer staging was determined based on histopathological findings in accordance with the 8th edition of the AJCC guidelines. Pathological evaluation included assessment of histological grade (well, moderately, or poorly differentiated), DOI, LVI, PNI, neck metastasis, WPOI, HPV test, and bone invasion.

### Statistical methods

The variables analyzed included gender, age, dental history, histological differentiation, primary tumor site, tumor stage, and DOI. Descriptive statistics and comparative analyses were performed using Microsoft Excel (Microsoft 365, Microsoft Corporation, Redmond, WA, USA) and IBM SPSS Statistics for Windows, version 22.0 (IBM Corp., Armonk, NY, USA). A two-sided exact binomial test was used to compare the distribution of OSCC between the mandible and maxilla. Fisher’s exact test was used to compare histologic differentiation by site. The Mann–Whitney U test was used to compare DOI between sites. Log-rank p-value was used to compare the significant difference in overall survival rate by site. A *p*-value < 0.05 was considered statistically significant.

## Result

### Patient characteristics

**Fig. 1:**
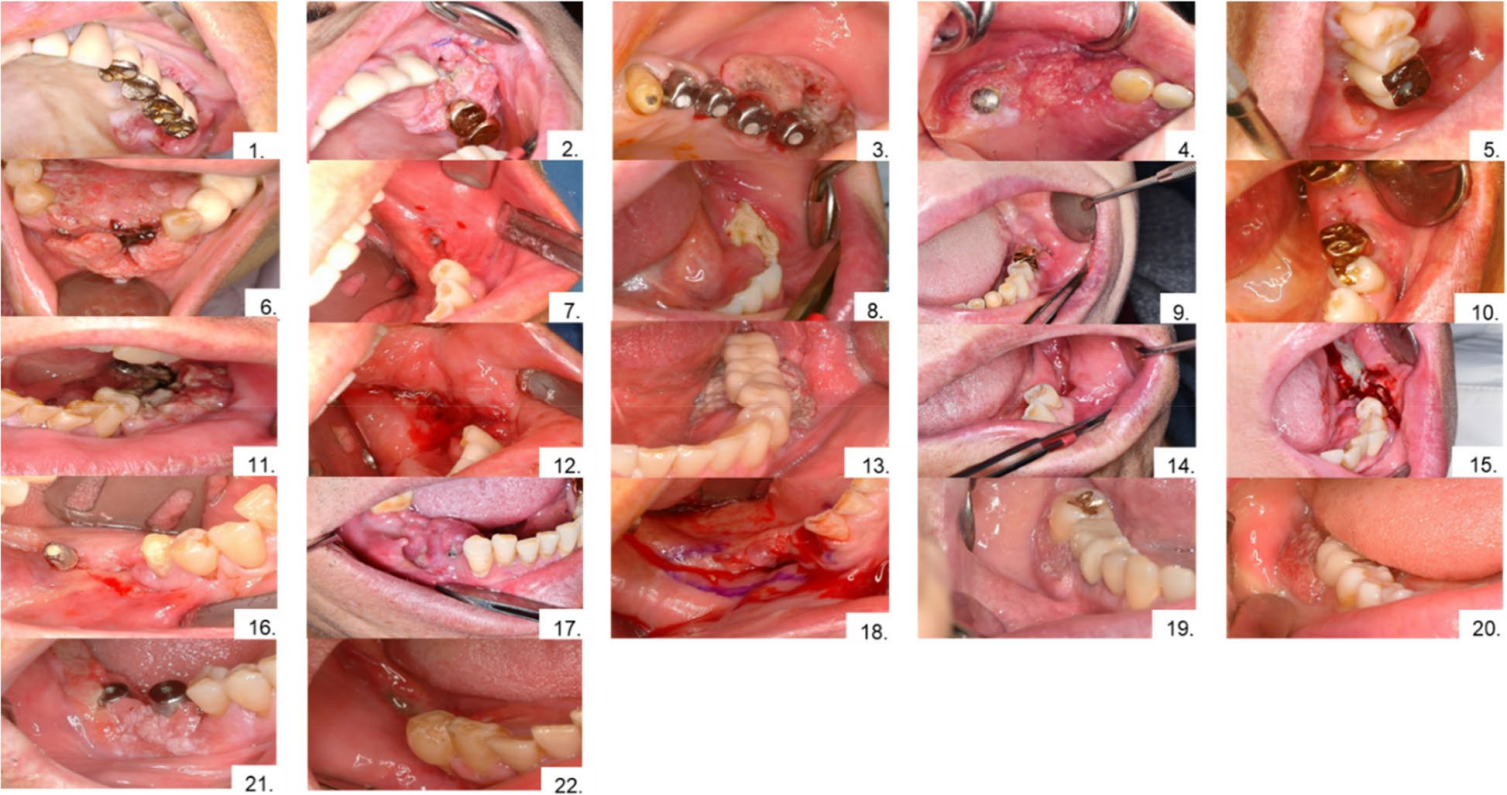
Intraoral photograph at first visit. OSCC arising around a dental implant, presenting as either an exophytic or ulcerative lesion

**Fig. 2:**
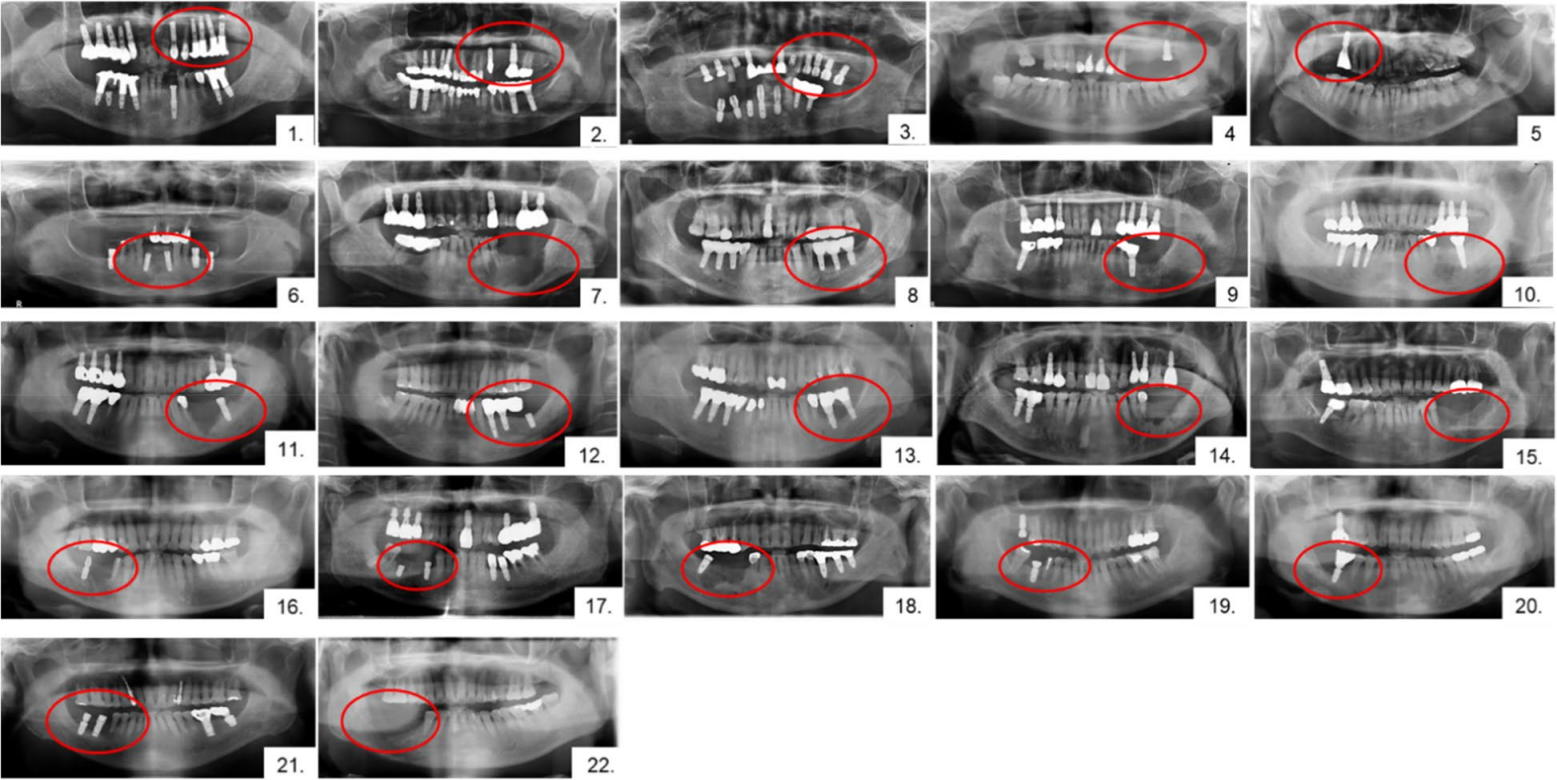
Panoramic radiograph at first visit. The red circle indicates the site of OSCC occurrence. Most cases showed marginal alveolar bone loss around the dental implant, whereas some cases (e.g., cases 1, 6, 12, 19) did not show definite bone loss

**Fig. 3:**
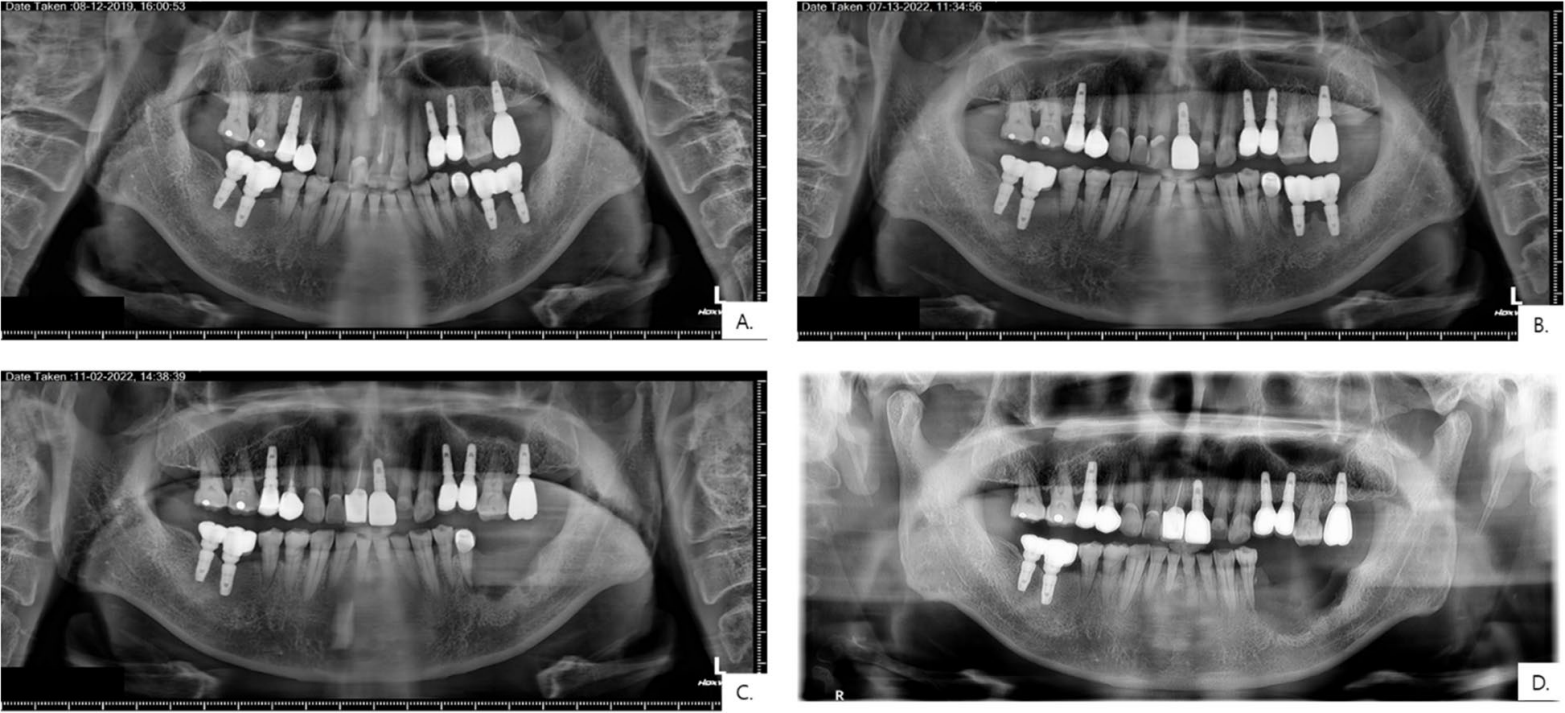
Serial panoramic radiographs of a patient before OSCC diagnosis. The patient underwent implant placement at the left mandible (#36, 37) in 2008 at a local clinic. **A **August 2019: marginal bone loss noted around #36, 37. (panoramic X-ray from the local clinic). **B **July 2022: progressive bone loss observed with discomfort (panoramic X-ray taken from the local clinic). **C** November 2022 (after implant removal): further bone loss encroaching #35. (panoramic X-ray from the local clinic). **D** February 2023 (Department of Oral and Maxillofacial Surgery): severe bone destruction; incisional biopsy confirmed OSCC following referral from the local clinic

All 22 patients presented with persistent pain with abnormal lesions or progressive bone loss around the dental implant (Figs. 1 and 2). OSCC occurred more frequently in the mandible (77.3%) than in the maxilla (22.7%) (binomial test, *p* = 0.017, Table 1). Five patients (22.7%) had a prior history of malignancy, and six male patients reported long-term smoking and alcohol consumption; they were advised to discontinue both behaviors, although cessation success was not assessed (Tables 1 and 2). The mean time from dental implant placement to OSCC diagnosis was 4.7 years, with nearly half diagnosed within 1 year and slightly more than half after > 1 year (Fig. 3).

**Table 1:** Raw demographic and clinical information of the patients

Patient no.	Gender	Age (at first visit)	OSCC site	Known PMH	Smoking/drinking (years)	Interval since implantation (years)	Treatment done at other institution before referral	Deceased (Lifespan)
1	M	82	Lt. Mx	HTN, stent	30/30	20	Observe	X
2	F	72	Lt. Mx	NS	−/−	N/A	Implant removal	X
3	M	81	Lt. Mx	Lt. Mx ca HTN,DM,CKD	−/−	< 1	Observe	O (7.2 months)
4	F	66	Lt. Mx	Bladder ca HTN,DM, osteoporosis	−/−	2	Implant removal	X
5	M	78	Rt. Mx	HTN,HL, BPH, gout	−/−	< 1	Observe	O (8.4 months)
6	F	78	Ant. Mn	HTN	−/−	2	Observe	X
7	M	80	Lt. Mn	Rectal Ca	−/−	5	Implant removal	X
8	F	56	Lt. Mn	Thyroidectomy due to thyroid nodule	−/−	< 1	Implant removal	X
9	F	66	Lt. Mn	NSCLC, thyroid ca(s/p total thyroidectomy), renal cell Ca	−/−	N/A	Implant removal	X
10	M	68	Lt. Mn	Vertebral artery stenosis	−/−	10	Implant removal	X
11	M	71	Lt. Mn	DM, CKD	25/25	N/A	Implant removal	O (19.3 months)
12	F	81	Lt. Mn	HTN	−/−	< 1	Observe	X
13	M	53	Lt. Mn	HTN	−/40	10	Observe	O (117.8 months)
14	M	81	Lt. Mn	HTN	50/−	15	Implant removal	O (18.4 months)
15	F	66	Lt. Mn	HTN,HL	−/−	10	Implant removal	O (8.7 months)
16	M	77	Rt. Mn	HTN,DM	30/60	< 1	Observe	O (24.9 months)
17	M	52	Rt. Mn	HTN	35/35	< 1	Implant removal	X
18	M	64	Rt. Mn	HTN	40/45	< 1	Implant removal	O (18.5 months)
19	F	55	Rt. Mn	NS	−/−	2	Observe	X
20	F	52	Rt. Mn	NS	−/−	5	Observe	X
21	M	82	Rt. Mn	Multiple myeloma	NA/NA	< 1	Observe	O (14.2 months)
22	M	51	Rt. Mn	NS	20/20	< 1	Implant removal	O (20.2 months)

*HTN *hypertension, *HL *hyperlipidemia, *DM *diabetes mellitus, *CKD *chronic kidney disease, *NS *not specified, *Ca *carcinoma, *NA *not available

**Table 2:** Summarized demographic and clinical information of the patients

Gender	
Male	13 (59.1%)
Female	9 (40.9%)
Age (years)	
< 65	7 (31.8%)
≥ 65	15 (68.2%)
Habitual alcohol consumption	6(27.3%)
Habitual smoker	6(27.3%)
Time between Dental implant and OSCC diagnosis	
< 1 year	9
> 1 year	10
Unknown	3
T-stage	
T1	1 (4.5%)
T2	0 (0.0%)
T3	0 (0.0%)
T4	21 (95.5%)
Neck metastasis	
Positive	9 (41.0%)
Negative	13 (59.0%)
DOI^a^	
< 5	1(5.6%)
≥ 5	17 (94.4%)
HPV	
Positive	1
Negative	10
Unidentified	11
Cell differentiation	
Well-differentiated	13
Moderately differentiated	9
Poorly differentiated	0

^a^Excluded DOI of four patients due to unidentified DOI

### Histopathologic findings

**Fig. 4:**
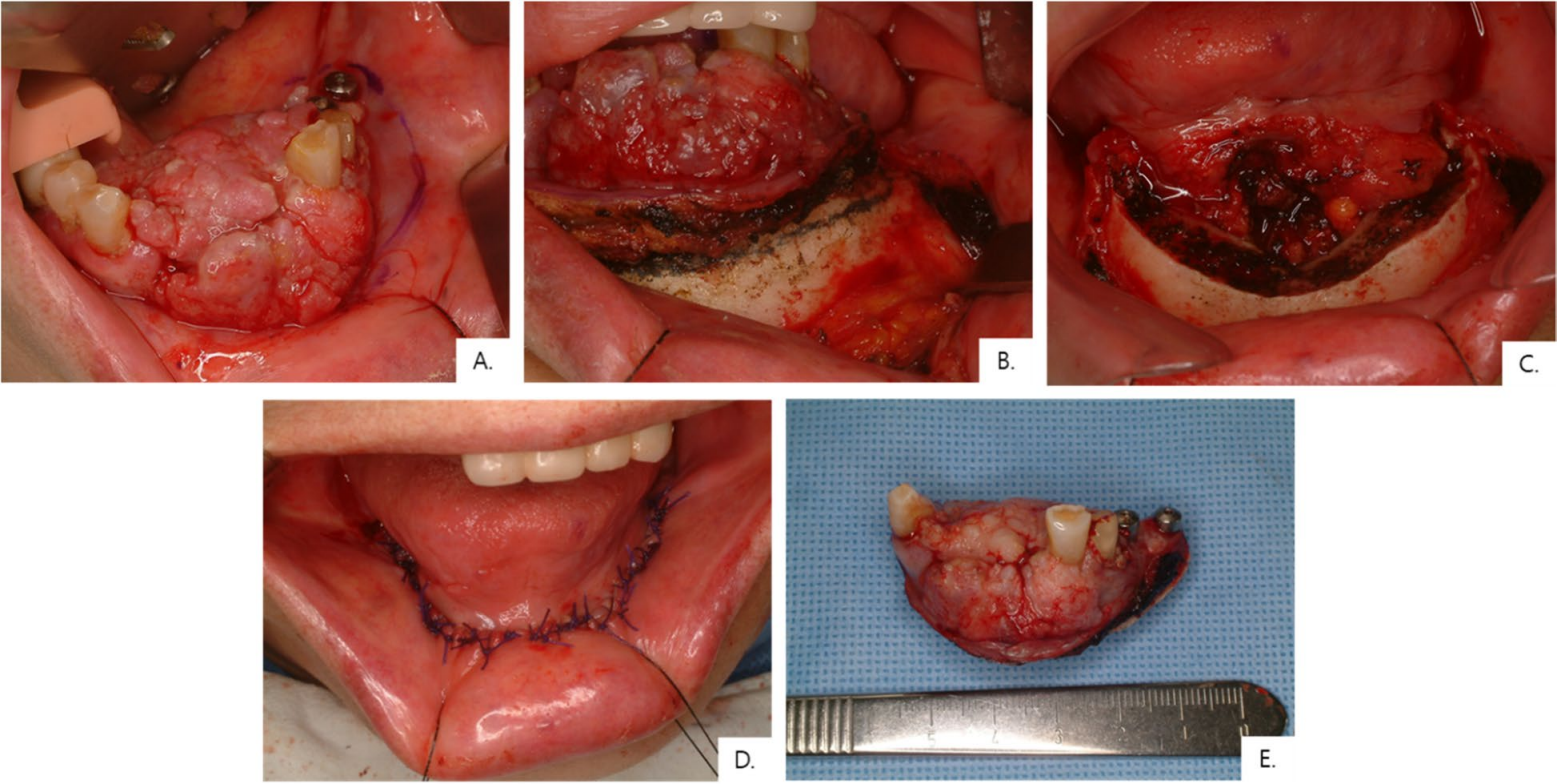
OSCC arising around a dental implant in the anterior mandible. **A **Preoperative intraoral photograph. **B** Clinical view before marginal mandibulectomy. **C** Post-mandibulectomy surgical field. **D** Primary closure following resection. **E** Final surgical specimen showing clear margins with a depth of invasion (DOI) of 5 mm. The patient had no tumor recurrence or nodal metastasis during follow-up

**Fig. 5:**
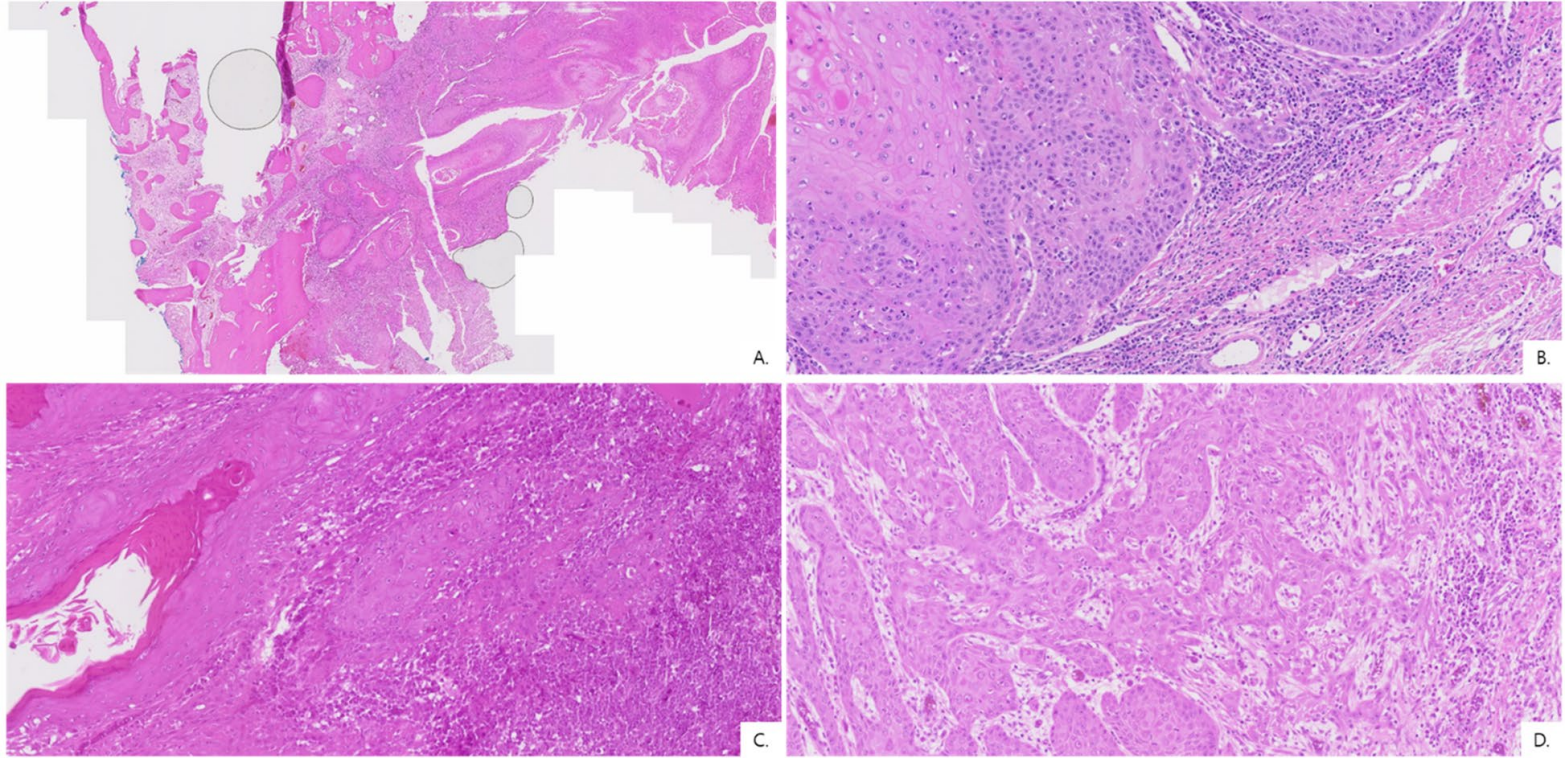
Histopathological examination of OSCC surgical specimens (H&E stain). Sections show islands of atypical squamous epithelial cells infiltrating the stroma with keratinization, keratin pearls, pleomorphism, hyperchromatic nuclei, and increased mitotic activity. **A** Well-differentiated SCC (H&E, × 1) .**B** Well-differentiated SCC (H&E, × 2). **C** Well-differentiated SCC (H&E, × 20) **D **Moderately differentiated SCC (H&E, × 20)

Most patients presented with advanced disease, with 95.5% classified as T4 at diagnosis (Table 3 and Fig. 4). Neck metastasis was observed in 41% of patients, including four with delayed/occult nodal metastases during follow-up (Table 4). Of the four delayed/occult cases, three occurred in the maxilla. Histopathologic analysis demonstrated that all tumors were either well (59%) or moderately (41%) differentiated, with no poorly differentiated cases (Fig. 5). Histologic differentiation did not differ significantly between the maxilla and mandible (Fisher’s exact test, *p* = 0.36, Table 4). Bone invasion was observed in all maxillary cases and in all but one mandibular case (95.5%) (Table 5). The mean DOI was 13.2 mm overall, and no statistically significant difference was found between the mandible and maxilla (Mann–Whitney *U* test, *p* = 0.729).

**Table 3:** Raw histopathologic information of the patients

Patient no.	DOI (mm)	TNM stage	PNI	LVI	WPOI	HPV	Neck metastasis	Cell differentiation	Bone invasion
1	9	cT2N0M0 pT4a	−	+	5	−	Occult meta	Moderately	+
2	16	cT4aN0M0 pT4a	−	+	5	NA	−	Well	+
3	8	cT4aN0M0 pT4a	−	−	1–4	−	Occult meta	Well	+
4	8	cT4aN0/2bM0 pT4a	−	−	1–4	NA	−	Well	+
5	7.5	cT4aN0M0 pT4a	+	+	5	NA	Delayed neck meta	Well	+
6	5	cT4aN0M0 pT4a	−	−	1–4	NA	−	Well	+
7	25	cT4aN1M0 pT4aN1	−	+	1–4	NA	Left Lv. IIa	Moderately	+
8	NA	NA pT4aN0	−	−	NA	−	−	Well	+
9	15	cT4aN2b/0M0 pT4aN0	−	+	5	−	−	Well	+
10	16	cT4aN0M0 pT4aN0	−	−	NA	+	−	Well	+
11	12	cT4aN2M0 pT4aN0	−	−	1–4	−	−	Well	+
12	NA	cT4aN0M0 pT4aN0	−	−	NA	−	−	Well	+
13	7	cT4aN0M0 pT4aN0	−	−	NA	NA	−	Moderately	+
14	24	cT4aN2b/0M0 pT4aN1	+	−	5	NA	Left Lv. IIa	Moderately	+
15	13	cT4aN2bM0 pT4aN3b	+	−	1–4	−	Left Lv. Ib, IIa,	Moderately	+
16	25	cT4aN0M0 pT4aN0	−	−	NA	−	−	Moderately	+
17^a^	NA	cT4aN1/N2bM1 NA	NA	NA	NA	−	−	Moderately	+
18	5	cT4aN0M0 pT4aN0	−	−	NA	−	Delayed neck meta	Well	+
19	2	cT2N0M0 pT1	−	−	NA	NA	−	Well	−
20	7	cT4aN0M0 pT4aN0	−	−	NA	NA	−	Moderately	+
21^b^	NA	cT4aN2bM0 NA	−	−	NA	NA	Right Lv. I, II	Well	+
22	38	cT4aN0M0 pT4aN1	−	+	NA	NA	Right Lv. II (occult metastasis)	Moderately	+

*HPV *human papillomavirus, *DOI* depth of invasion, *PNI* perineural invasion, *LVI *lymphovascular invasion, *WPOI*, *NA *not available

^a^The surgery was not done due to lung metastases were detected during initial cancer staging work-up

^b^The surgery was not done due to poor general condition

**Table 4:** Histopathologic characteristics of OSCC categorized by maxilla and mandible

Location (*n*)	Mean DOI (mm)	Cell differentiation	Neck metastasis
Mx (*n* = 5)	9.7	Well: 4 Moderately: 1 Poorly: 0	3(3^a^)(60.0%)
Mn. (*n* = 17)	14.9*	Well: 9 Moderately: 8 Poorly: 0	6(1^a^)(35.3%)

^a^Patients who experienced delayed/occult neck metastasis

*DOI *depth of invasion, *PNI *perineural invasion, *LVI *lymphovascular invasion, *Mx* Maxilla, *Mn* mandible

^*^Excluded DOI of four patients due to unidentified DOI

**Table 5:** Histopathologic characteristics of OSCC categorized by area

Location (*n*)	Mean DOI (mm)	LVI( +)	PNI( +)	Neck metastasis	Bone invasion
Lt. Mx (*n* = 4)	10.3	2(50%)	0	2(2^a^)(50%)	4(100%)
Rt. Mx (*n* = 1)	7.5	1(100%)	0	1(1^a^)(100%)	1(100%)
Lt. Mn (*n* = 9)	16*	2(22.2%)	2(22.2%)	3(33.3%)	9(100%)
Rt. Mn (*n* = 7)	15.4*	2(28.6%)	2(28.6%)	3(1^a^)(42.9%)	6(85.7%)
Ant. Mn (*n* = 1)	5	0	0	0	1(100%)

^a^Patients who experienced delayed/occult neck metastasis

*DOI* depth of invasion, *PNI* perineural invasion, *LVI* lymphovascular invasion, *Mx* maxilla, *Mn* mandible

^*^Excluded DOI of four patients due to unidentified DOI

### Survival analysis

**Fig. 6:**
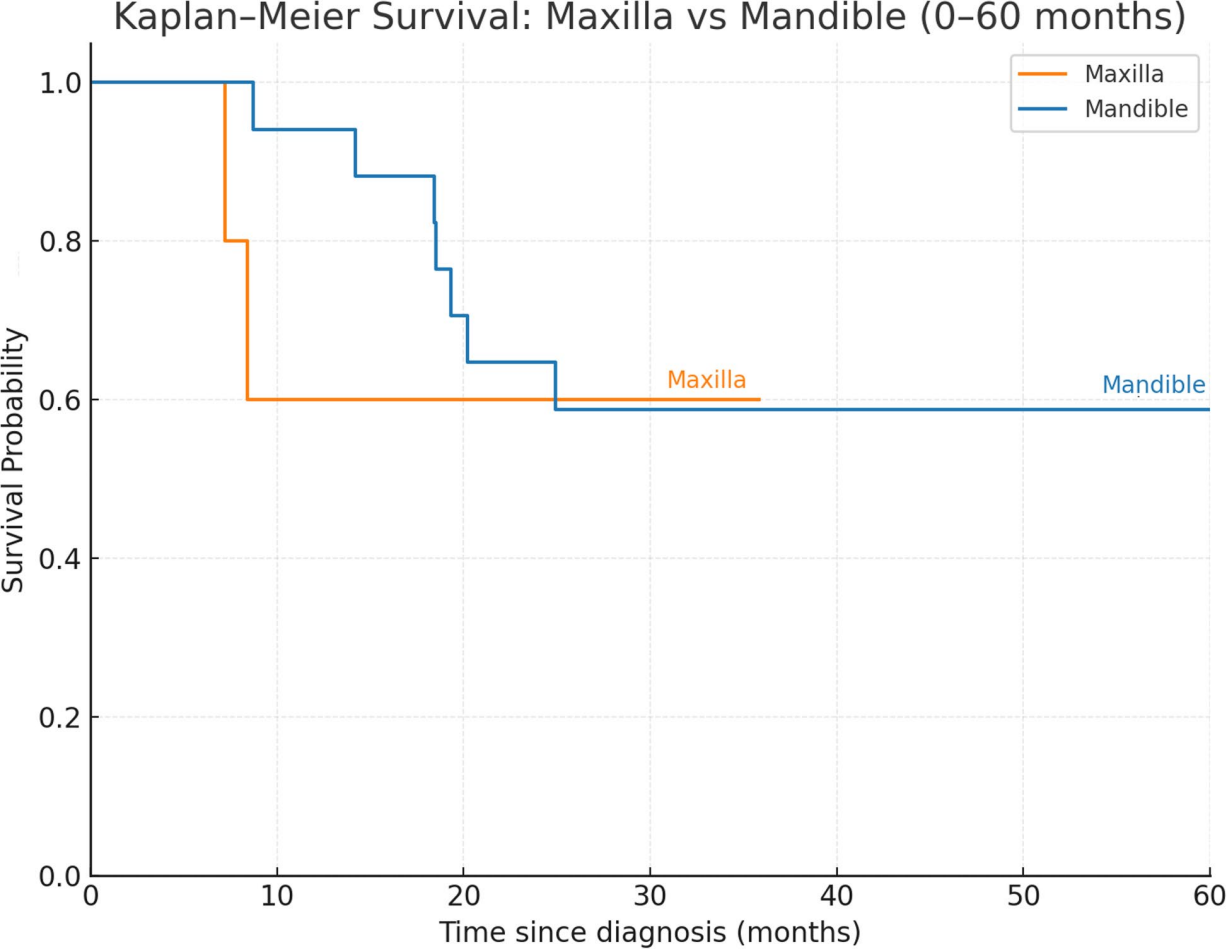
Kaplan–Meier survival curve for OSCC around dental implant in maxilla and mandible maxilla (*n* = 5): Median overall survival was not reached; the observed 1- and 3-year overall survival rates were both 60.0% (95% CI 0.17–1.00). Mandible (*n* = 17): median overall survival = 116.4 months; 1-year OS 94.0% (95% CI 0.83–1.00); 3-year OS: 54.0% (95% CI 0.29–0.79)

The mean overall survival time was 25.7 months, with a mean follow-up of 35.9 months (range, 3.84–125.6 months). Kaplan–Meier analysis showed no statistically significant difference in overall survival between maxillary and mandibular OSCC (log-rank, *p* = 0.36, Fig. 6), with 1- and 3-year overall survival of 94% and 54% for mandibular cases.

## Discussion

The mechanisms by which dental implants may contribute to OSCC remain unclear. Chronic peri-implant inflammation—caused by trauma, infection, or poor hygiene—promotes carcinogenesis by inducing oxidative stress, DNA damage, and cytokine-mediated tissue injury [5, 19, 20]. Reactive oxygen and nitrogen species further impair genomic integrity, activate oncogenic pathways, and increase resistance to apoptosis, favoring malignant transformation [21]. Clinical reports consistently associate chronic peri-implant inflammation with OSCC [22–24]. Rigorous plaque control, patient education, and regular professional care are essential to reduce peri-implant inflammation and cancer risk [25, 26].

Titanium hypersensitivity, though rare, can aggravate inflammation and act as a cofactor in carcinogenesis. Most implants use Ti-6Al-4 V, which is biocompatible and has a protective TiO₂ layer [27]. However, acidic pH, biofilms, and oxidative stress may disrupt this layer, leading to corrosion and ion release [28–30]. Surface modifications like sandblasting or acid etching can leave defects that accelerate corrosion under inflammation. Released titanium ions and particles are cytotoxic, promoting peri-implant disease and, in susceptible patients, malignant transformation [24, 31–34]. Hypersensitivity reactions (≈ 0.6% prevalence) may manifest as Type I or IV responses, contributing to peri-implantitis, chronic mucosal inflammation, and occasionally epithelial dysplasia [35–37]. Ill-fitting prostheses and excessive occlusal forces can perpetuate trauma-induced inflammation, increasing cancer risk [23].

Despite the high prevalence of peri-implantitis, only a minority of patients develop OSCC, suggesting a genetic predisposition. Patients with prior malignancy may carry mutations in tumor suppressors (e.g., TP53) or DNA repair genes [38, 39]. TP53 mutations, present in over half of OSCC cases, are linked to poor prognosis [40]. Variants in VAV2 and IQGAP1, involved in inflammatory signaling and epithelial integrity, have also been associated with familial OSCC [39]. These genetic vulnerabilities lower the threshold for neoplastic change under chronic DI-related irritation. Oncogenic pathways such as Wnt/β-catenin and RAS contribute similarly to OSCC [41, 42]. Peri-implant inflammation may activate NF-κB and STAT3 signaling, promoting survival, immune evasion, and tumor growth [19, 43]. Immunosuppression or chemotherapy can prolong mucosal injury, further increasing oncogenic risk.

Alcohol and tobacco are well-established synergistic risk factors that impair healing, generate reactive species, and worsen inflammation [19, 44–46]. In our study, 27.3% of patients—exclusively male—reported long-term use of both (Tables 1 and 2). Notably, 22.7% of patients with prior malignancy but without these habits also developed OSCC, indicating multifactorial etiologies. Overall, half of the cohort had at least one major risk factor (prior malignancy, alcohol, or tobacco), highlighting the need for close surveillance upon dental implant surgery.

Notably, the interval from the onset of peri-implant symptoms to definitive OSCC diagnosis ranged from 3 months to 1 year, likely contributing to the advanced stage at presentation and underscoring the diagnostic challenges as early OSCC often mimics peri-implantitis. Furthermore, twelve patients were referred to our clinic following implant removal performed at other hospitals, where abnormal healing patterns or persistent lesions raised suspicion and prompted further investigation. Figure 3 illustrates a representative case showing this latency. The patient received dental implant placement in 2008 at a local clinic, developed radiographic peri-implant bone loss by 2019, and reported clinical discomfort starting in July 2022, leading to eventual OSCC diagnosis. This timeline highlights the potential for a prolonged subclinical phase of peri-implant disease before malignant transformation becomes clinically apparent, emphasizing the need for routine long-term monitoring even years after implant placement. Persistent peri-implant lesions unresponsive to therapy should therefore prompt biopsy within 2–4 weeks [47–49]. Three cases were diagnosed as primary intraosseous carcinoma (PIOC), a rare malignancy of the jawbone, possibly linked to chronic inflammation [50–52]. High-risk patients should undergo regular follow-up with cone beam computed tomography (CBCT) imaging and biopsy for progressive bone loss.

Demographically, our cohort had a mean age of 68.7 years, with 68.2% over 65, consistent with age-related OSCC risk [1, 53]. Male predominance (59.1%) likely reflects behavioral exposures [1, 54]. Nearly half of cases occurred within 1 year of DI placement, while the remainder presented up to 20 years later, indicating variable latency. HPV was detected in only one of 11 patients, reinforcing that implant-associated OSCC is primarily inflammation-driven rather than virally mediated [55].

Mandibular involvement predominated (77.3%), significantly more frequent than maxillary cases (*p* = 0.017). Bone invasion occurred in 95.5% of tumors, exceeding previously reported rates (12–56%) [56–58]. The mandible’s greater susceptibility may relate to thinner mucosa, higher bone density, and implant biomechanics [58–61].

Prognostically, DOI is a key indicator. For TNM classification, DOI, included in AJCC 8th edition staging, correlates with nodal metastasis, recurrence, and mortality [17, 18, 62, 63]. In our cohort, 95.5% of tumors were T4 with bone invasion; 27.3% had nodal metastasis at diagnosis, and 18.2% developed delayed/nodal metastasis. Mean DOI was 13.2 mm, higher in mandibular tumors (14.9 mm) than maxillary (9.7 mm), though not statistically significant (Table 4). Eighteen tumors exceeded 5 mm DOI, indicating aggressive behavior of OSCC around the dental implant. Thus, the implant–bone interface may facilitate direct tumor spread [64–66]. Most tumors were well-differentiated (59.1%) or moderately differentiated (40.9%), with no poorly differentiated cases, consistent with prior reports [67]. No significant group differences were observed (*p* = 0.36).

In our series, cervical metastasis occurred in 3 of 5 maxillary OSCC cases, and all were delayed/occult metastases (Tables 3 and 4). Although the study comprises a relatively small number of maxillary OSCC cases, this rate appears higher than what is usually reported, as the frequency of cervical node involvement in maxillary OSCC has traditionally been considered as low as 22% [67]. The discrepancy suggests that T4 maxillary tumors, despite their local anatomical confinement in the early stages, may possess a higher propensity for occult nodal spread than previously assumed. This finding underscores the need for elective neck dissection for dental implant–associated maxillary OSCC, given that delayed/occult neck metastasis may develop during follow-up.

Despite a mean follow-up of 35.9 months, survival was poor, with 10 deaths and a mean survival of 25.7 months (Fig. 6). The high rate of T4 disease may reflect a combination of bone invasion, delayed recognition of malignancy, and aggressive implant removal procedures initially undertaken for presumed peri-implantitis. Such delays and repeated surgical manipulation could contribute to tumor progression and likely account for the poor survival observed in this study. These findings indicate that more aggressive primary surgical management, such as wider excision margins and the elective neck dissection, may be warranted in cases of OSCC arising around dental implants.

This study is limited by its small sample size and relatively short follow-up. Larger multicenter studies with standardized histopathological protocols are needed to validate our findings. Due to the lack of detailed information on implant model and manufacturer, as well as crown types, it was not possible to evaluate the potential role of implant surface characteristics or modifications in the pathogenesis of OSCC. Although dental implants are not intrinsically carcinogenic, they can promote chronic inflammation and local trauma, potentially lowering the threshold for oncogenesis in susceptible patients. While peri-implantitis and chronic periodontitis are common pathways of chronic mucosal inflammation, implant-associated OSCC may involve additional factors, including titanium ion release, implant placement technique, and prosthetic design, which distinguish it from periodontitis-related carcinogenesis. Recognizing these differences is essential for developing surveillance and preventive strategies in both implant and tooth-retaining patients. Although cancer risk is not routinely discussed during implant counseling, clinicians should consider addressing it in patients with high-risk profiles. Emerging approaches, such as anti-inflammatory surface coatings of dental implants, may provide future preventive benefits. Importantly, persistent peri-implant inflammation warrants timely biopsy, and high-risk patients should be monitored through structured follow-up programs.

Three key lessons emerge from this case series: (1) persistent peri-implant inflammation should not be presumed benign—biopsy or referral to a higher-level care facility is warranted if symptoms persist beyond weeks; (2) patients with prior malignancy, tobacco, or alcohol exposure need closer surveillance after DI placement; and (3) early recognition is crucial to prevent advanced-stage diagnosis. Future studies should examine whether implant-associated OSCC has distinct histological or molecular characteristics compared with other OSCC subtypes.

## Conclusion

Although OSCC following dental implant placement is rare, it remains a major clinical concern due to delayed diagnosis, aggressive invasion, and poor prognosis. This study highlights the clinical and histopathological features of implant-associated OSCC, emphasizing its frequent advanced-stage presentation and strong bone invasion tendency. Early differentiation from benign peri-implant disease is crucial, as OSCC can mimic peri-implantitis; biopsy should be pursued when inflammation fails to resolve with standard therapy. Timely detection is essential to improve outcomes. The associations with prior malignancy, chronic inflammation, and behavioral risk factors indicate a multifactorial etiology. Larger longitudinal studies are needed to clarify genetic, environmental, and immunological contributors and to refine preventive and diagnostic strategies in implant dentistry.

## Data Availability

No datasets were generated or analyzed during the current study.

## Abbreviations

OSCC: Oral squamous cell carcinoma
DI: Dental implant
AJCC: American Joint Committee on Cancer
HPV: Human papillomavirus
DOI: Depth of invasion
PNI: Perineural invasion
LVI: Lymphovascular invasion
WPOI: Worst pattern of invasion
PIOC: Primary intraosseous carcinoma
Mx: Maxilla
Mn: Mandible
MRI: Magnetic resonance imaging
CT: Contrast-enhanced computed tomography
PET/CT: Positron emission tomography/computed tomography
CBCT: Cone beam computed tomography
HTN: Hypertension
HL: Hyperlipidemia
DM: Diabetes mellitus
CKD: Chronic kidney disease
NS: Not specified
Ca: Carcinoma
NA: Not available
